# CSF sTREM2 is associated with distinct microglial responses with separate effects on Alzheimer's pathology in the disease's trajectory

**DOI:** 10.1002/alz70856_100766

**Published:** 2025-12-25

**Authors:** Marie Emilie Tuil, Bshaier Allehyany, Rifa Sanjida Punnota, Paul Edison

**Affiliations:** ^1^ Imperial College London, London, United Kingdom; ^2^ Imperial College London, London, Greater London, United Kingdom; ^3^ Division of Neurology, Department of Brain Sciences, Imperial College London, United Kingdom, London, London, United Kingdom

## Abstract

**Background:**

Microglial activation is an important element of AD pathology, yet its role remains poorly understood. There is significant evidence that microglia contribute to neuroinflammation and neurotoxicity in AD^1,2^, yet there is also evidence to the contrary. TREM2 loss‐of‐function mutations, an important risk factor, depress the microglial response^3^, while CD163+TREM2‐expressing amyloid‐reactive microglia (ARM) appear neuroprotective^4^. This suggests several distinct microglial responses, possibly stage and region‐specific.

We evaluated the associations between AD biomarkers and cognition to understand the impact of the microglial response on pathology throughout AD trajectory.

**Method:**

323 participants, with CSF sTREM2, GAP‐43, Aβ_1‐42_, T‐tau, and *p*‐tau_181_, MRI, 18F‐AV45, 18F‐FDG scans, and ADAS‐Cog‐13 scores, were selected from the ADNI database (https://adni.loni.usc.edu/). They were stratified into: Aβ+AD (*n* = 89), Aβ+MCI (*n* = 140), and Aβ−MCI (*n* = 94). We studied the relationships between biomarkers and cognition using Pearson correlations.

**Result:**

TREM2 shows very significant (*p* <0.001) associations with *p*‐ and t‐tau in both MCI groups (r=0.618 and 0.615 in Aβ‐MCI, and r=0.507 and 0.477 in Aβ+MCI) yet none in AD. TREM2 also shows a very significant association (*p* <0.001) with Aβ_1‐42_ in Aβ‐MCI (r=0.401), and significant associations in Aβ+MCI and AD (r=0.203, *p* = 0.016; r=0.303, *p* = 0.004). Finally, Aβ_1‐42_ is associated with GAP43 in Aβ‐MCI and AD (r=0.304, *p* = 0.003; r=0.218, *p* = 0.040) but not in Aβ+MCI, at which stage we observe the only negative association between amyloid and ADAS_Cog13 (r=‐0.263, *p* = 0.002).

**Conclusion:**

TREM2 associations suggest distinct and diverging stage‐dependent microglial responses in AD, with a neurotoxic Aβ‐dependent response in Aβ‐MCI. In Aβ+MCI, the microglial response is associated with preserved cognition, suggesting a neuroprotective phenotype has emerged in response to high amyloid load. In AD, the response turns neurotoxic again as pathology continues to accumulate.

**References**

1. Heneka MT, et al. Neuroinflammation in Alzheimer disease. *Nat Rev Immunol*. Dec 9 2024;doi:10.1038/s41577‐024‐01104‐7

2. Boche D, Nicoll JAR. Invited Review ‐ Understanding cause and effect in Alzheimer's pathophysiology: Implications for clinical trials. *Neuropathol Appl Neurobiol*. Dec 2020;46(7):623‐640.doi:10.1111/nan.12642

3. Valiukas Z, et al. Microglial activation states and their implications for Alzheimer's Disease. *J Prev Alzheimers Dis*. Jan 2025;12(1):100013.doi:10.1016/j.tjpad.2024.100013

4. Nguyen AT, et al. APOE and TREM2 regulate amyloid‐responsive microglia in Alzheimer's disease. *Acta Neuropathol*. Oct 2020;140(4):477‐493.doi:10.1007/s00401‐020‐02200‐3

